# Accurate extraction of electrical parameters in three-diode photovoltaic systems through the enhanced mother tree methodology: A novel approach for parameter estimation

**DOI:** 10.1371/journal.pone.0318575

**Published:** 2025-03-04

**Authors:** Mouncef El Marghichi, Abdelilah Hilali, Abdelkhalek Chellakhi, Mohamed Makhad, Azeddine Loulijat, Najib El Ouanjli, Abdelhak Essounaini, Vikash Kumar Saini, Ameena Saad Al-Sumaiti

**Affiliations:** 1 Intelligent Systems Design Laboratory (ISD), Faculty of Science, Abdelmalek Essaadi University, Tetouan, Morocco; 2 Faculty of Sciences, Moulay Ismail University, Meknes, Morocco; 3 Laboratory of Engineering Sciences for Energy (LabSIPE), National School of Applied Sciences of El Jadida, Chouaib Doukkali University, El Jadida, Morocco; 4 Departement of Electrical Engineering, ENSAM Rabat. Mohammed V University, Rabat, Morocco; 5 Faculty of Sciences and Technology, Hassan First University, Settat, Morocco; 6 Electrical Engineering Department, Higher School of Technology, Moulay Ismail University, Meknes, Morocco; 7 Laboratory of Analysis, Modeling and Simulation, Department of Mathematics and Computer Science, Faculty of Sciences Ben M’Sik Sidi Othman, Hassan II University, Casablanca, Morocco; 8 Smart OR Lab, Advanced Power and Energy Centre, Department of Electrical Engineering, Khalifa University, Abu Dhabi, UAE; Vellore Institute of Technology, INDIA

## Abstract

Accurately simulating photovoltaic (PV) modules requires precise parameter extraction, a complex task due to the nonlinear nature of these systems. This study introduces the Mother Tree Optimization with Climate Change (MTO-CL) algorithm to address this challenge by enhancing parameter estimation for a solar PV three-diode model. MTO-CL improves optimization performance by incorporating climate change-inspired adaptations, which affect two key phases: elimination (refreshing 20% of suboptimal solutions) and distortion (slight adjustments to 80% of remaining solutions). This balance between exploration and exploitation allows the algorithm to dynamically and effectively identify optimal parameters. Compared to seven alternative methods, MTO-CL shows superior performance in parameter estimation for various solar modules, including ST40 and SM55, across different irradiances and temperatures. It achieves exceptionally low Root Mean Square Error (RMSE) values from 0.0025A to 0.0165A and Mean Squared Error (MSE) values between 6.2 × 10^−6 and 2.7 × 10^−4, while also significantly minimizing power errors, ranging from 22.86 mW to 239.40 mW. These results demonstrate MTO-CL’s effectiveness in improving the accuracy and reliability of PV system modeling, offering a robust tool for enhanced solar energy applications.

## 1. Introduction

Human life’s sustainability is intricately linked to enhanced life quality which necessitates power resources and progress in sustainable energy. The decline of traditional energy reservoirs has resulted in environmental degradation. As a result, a crucial pivot towards sustainable energy supplies becomes unavoidable. These energy sources boast cleanliness, address environmental concerns, are abundant, and exhibit versatility in various applications [1,2].

In recent times, solar and wind power, along with other sustainable energy sources, have witnessed remarkable economic value [3,4], progress, and applications [5], demonstrating improved effectiveness in energy production [6]. These environmentally friendly and long-lasting energy alternatives have taken center stage in research pursuits. Notably, solar photovoltaic (PV) technology has become a significant contributor to various applications, ranging from to water demineralization and heating/cooling systems [7]. With their variant nature [8], achieving exact solar cell simulation and modeling entails employing different methodologies, including numerical simulation [9] and adaptive control [10].

Solar cells exploit a semiconductor material characterized by a P-N junction with differentiated regions:1) quasi-neutral, 2) space-charge,and 3) defect regions, experiencing losses as a matter of charge transporter diffusion and recombination [11]. It is imperative to consider these losses when developing a PV model. In an ideal model, the photovoltaic cell produces a photo-generated current, deviating of the actual PV model’s measured value, as a result of P-N junction losses in the semiconductor. Accordingly, the PV model employs Single Diode Models (SDMs), renowned for their simplicity and speed in expressing losses in the near-neutral region [11]. To enhance precision, Double Diode Models (DDMs) are utilized, accounting for losses in SDM and space charge regions. In addition, the Three Diode Models (TDMs) are implemented for heightened precision, including defect region losses and those delineated in DDM [11]. A precise model is needed for deploying PV systems, imposing accurate estimating of the parameters of the PV cells model [12]. Yet, due to the nonlinearity and non-convexity characteristics of the PV model, numerous challenges are encountered. In response, the literature have devised three distinct techniques to precisely estimate parameters: analytical, determinist and metaheuristic approaches [13].

Analytical techniques depend on particular data points (e.g.,open and short circuit data) to formulate straightforward mathematical models for dealing with model parameters. While these techniques are efficient and user-friendly, their efficacy heavily relies on the precision of the data supplied by manufacturers, potentially compromising accuracy. Additionally, the accuracy of these techniques may be susceptible to degradation over time in photovoltaic (PV) systems [14].

The various numerical methods are explained in historical study for dynamic performance analysis of PV units. Rung-Kutta optimizer (RKO) approach is deployed for extracting the optimal unknown parameters of the TDM. Further, RKO results undergo comparison with various other recognized optimizers. The purpose of these comparisons is to demonstrate the effectiveness and viability of the RKO [15]. The steepest descent method, i.e., Newton’s method, is the damped least squares method, which is commonly employed to solve nonlinear systems of equations [16]. However, these techniques rely on sequential information and suffer from shortcomings such as low sensitivity to noise, algorithmic complexity, and accuracy. Additionally, the selection of iteration steps requires operator expertise in numerical analysis. To address these issues, metaheuristic optimization algorithms have been introduced in various engineering applications.

The No Free Lunch (NFL) theorem [17] underscores the absence of a universal metaheuristic optimization technique capable of solving every problem in optimization. NFL emphasizes on the efficacy of an optimization algorithm on one group of tasks would not ensure similar results on another group of tasks. Widely acknowledged in the field, the NFL is used as a foundational concept for numerous studies, which empowers researchers to tailor available methods to address novel problem classes.

The performance of the solar PV modules is reliant on the value of module’s parameter, so the optimal value of parameters plays a vital role. The Wild Horse Optimizer (WHO) has been considered for extracting the parameters of DDM and TDM PV models in the reference [18]. It provides a discussion of the mathematical model and optimization problem of PV models; the aim is an accurate prediction of the electrical parameters of these models. The WHO is presented as a viable approach for accurate estimation of both dynamic and static PV models. Reference [19] presents the learning models that ensure reliable and accurate estimates of PV system parameters. The utilization of the gradient-based optimizer (GBO) to determine the parameters of SDM, DDM, and TDM PV models [20]. For accurate modeling of PV modules, in particular the three-diode model, [21] uses the Sunflower Optimization (SFO), reporting an error rate of less than 0.5% in all experiments. In [22] proposes an improved JAYA algorithm for robust and accurate simulation of solar cell voltage and current properties, outperforming other algorithms. An improved whale optimization algorithm is used in [23] for parameter estimation in DD and SD PV models, as well as PV module models, validating the models using experimental and simulation data. The Comprehensive Learning Jaya (CLJAYA) algorithm is used for extracting parameters from PV models [24], while [25] applies the Warfare Strategy Optimization (WSO) of the Floating Solar Photovoltaic (FSPV) SDM. The comparative study of various optimization algorithms is presented for solar PV module parameter estimation [26].

Moreover, a recent study [27] has provided a new perspective with the emergence of the Golden Search Optimization Algorithm (GSO), highlighting its potential for discerning the parameters of PV models. The GSO uses a highly efficient strategy to solve complicated problems, through initial random solutions dynamically adjusting to attain a global optimal solution. It is a global approach that combines exploitation and exploration. Compared with other Optimization strategies, GSO’s proven accuracy makes it an exceptional tool for precise parameter identification, control and optimization of photovoltaic systems.

In [28], a novel physics-based methodology termed the Resistance-Capacitance Optimization Algorithm (RCoA) is introduced for PV model parameters parameter extraction. It specifically suggests modifications to photovoltaic models, with a focus on triple-diode models. The RCoA algorithm draws inspiration from the feedback mechanism inherent in resistance-capacitance (RC) circuits, presenting itself as a promising technique for precisely estimating both static and dynamic photovoltaic (PV) models [29]. Similarly, other optimizers have been utilized for PV model parameters parameter extraction, i.e., Nutcracker Optimization [30], Northern Goshawk Optimization [31], atomic orbital search [32], Anti-sine-cosine atom search optimization (ASCASO) [33], CUCKOO Search Optimization (CSO) [34], Modified Bald Eagle Search Optimization (MBESO) [35], and hybrid PSO [36].

The Enhanced Artificial Gorilla Troops (EAGT) optimizer has been employed for extracting the parameter of a triple-diode model of PV systems [37]. This advanced intelligent algorithm is specifically crafted for effective parameter extraction within the realm of photovoltaic modeling. The study assesses the performance of the EAGT optimizer in comparison to other existing optimization methods and evaluates its efficacy in precisely estimating the electrical parameters of the photovoltaic triple-diode model. In [38], the fractional order Harris Hawks optimization algorithm is used to estimate the parameter of the photovoltaic triple diode model. The photovoltaic model and the optimization problem, aiming to accurately estimating the electrical parameters of the model. The TDM commercial solar PV model parameter has been extracted using the Berndt-Hall-Hall-Hausman as illustrated in [39].

Extraction of unknown parameters from PV cells is of vital importance for various applications, including maximum power point tracking (MPPT), fault diagnosis, control systems, and overall system performance analysis. These parameters include photocurrent, series and shunt resistance, diode ideality factor and reverse saturation current. Reference [40] employs the Chaotic War Strategy Optimization (CWSO) which aims to minimize the error among measured and true values in the parameter extraction process for SD, DD, and TD models.

For enhancing the PV module parameter, the White Shark Optimizer (WSO) includes the adjustment of force control parameters within the WSO, as well as the integration of a chaos generator to enhance its exploitation capabilities [41]. The resulting modified algorithm is denoted as IWSO and is employed for the extraction of PV parameters.

Recent efforts have focused on parameter estimation for solar PV modules and cell, employing both analytical and metaheuristic techniques. Analytical techniques narrow down the parameters determined by analyzing specific data, but despite requiring fewer computational resources, ensuring their accuracy is challenging.

A novel approach known as the Level-Based Learning Swarm Optimizer (LLSO) is introduced in [42]. This method draws inspiration from blended learning, a pedagogical strategy in which educators customize their methods according to the individual abilities of students. The LLSO utilizes historical knowledge and social cues within learning search algorithms for guiding the search process, effectively improving the global exploitation capabilities. Nevertheless, it enhances the overall learning efficiency of the population by integrating teaching as well as active learning according to optimal individuals, ultimately strengthening local development capabilities [43]. Reference [44] provides employed, please refer further explanations on the diverse learning algorithms [44]. The optimize deep neural network are presented in [45] for solar PV module parameter identifications. For balancing the algorithm’s exploration and development abilities, simple reinforcement learning is introduced, which is regulated and takes advantage of the benefits of CPA and NMS strategies via reward and punishment mechanisms [46].

In the domain of solar cell parameter estimation using metaheuristics, several challenges have been identified, including nonadaptive weight measurements, slow calculation rates, issues with local optima, and the need to reduce Root Mean Square Error (RMSE) values. Existing methods such as analytical techniques, while efficient, depend heavily on precise manufacturer data, which can compromise accuracy as PV systems age. Numerical methods, including Rung-Kutta optimizers (RKO) and Newton’s method, offer improvements but face issues such as sensitivity to noise, algorithmic complexity, and the need for operator expertise in choosing iteration steps. Metaheuristic approaches like WHO, GSO, and CLJAYA have made progress by providing more robust solutions, but they still encounter challenges related to local optima and computational speed.

The Mother Tree Optimization with Climate Change (MTO-CL) algorithm addresses these gaps by incorporating dynamic adaptation to changes in the search space, balancing exploration and exploitation more effectively. Inspired by the No Free Lunch (NFL) theorem, MTO-CL improves parameter estimation accuracy by integrating periodic climate change simulations to better model real-world variations in PV systems. This approach achieves globally optimal values with fewer iterations, demonstrating both efficiency and speed. Despite these advantages, MTO-CL still faces challenges with slower computation speeds compared to other methods. Future research should focus on enhancing its computational efficiency while maintaining high accuracy to ensure its effectiveness in practical applications [47].

This paper makes several notable contributions, which are highlighted as follows:

- This study introduces an efficient searching approach for accurate parameter estimation of PV cells, aiming at overcoming the current limitations of current estimation techniques, besides offering an innovative approach in the PV field. The MTO-CL algorithm is applied to achieve efficient parameter estimation. This novel computational metaheuristic employs two key phases, elimination and distortion, ensuring a balanced trade-off among exploration and exploitation in estimating solar PV parameters.
- The distinctive feature of MTO-CL lies on how dynamically it adapts to temperature and irradiation changes, facilitating extracting PV parameters with maximum effectiveness. The solid algorithm performance is clear in its accuracy, consistency and creating smooth balance between exploitation and exploration in parameters optimization.

MTO-CL is developed according to the principles of the No Free Lunch (NFL) theorem, building upon its foundation. The successful accomplishment in addressing the PV parameters’ estimation problem through this algorithm strengthens its feasibility for real application.

- Seven robust algorithms are compared with the MTO-CL algorithm as a reference. The results of this analysis confirm the efficiency and resilience of the MTO-CL algorithm, making it the preferred choice for parameter estimation in photovoltaic models.
- Despite the exceptional accuracy of the proposed solution, it is essential to note that this increased accuracy comes at the expense of speed. Extensive experiments with real solar modules (ST40 and SM55) have confirmed its accuracy; however, it takes longer to obtain optimal overall results in comparison with other algorithms. Indeed, improving the speed of the suggested algorithm will be the focus of future work.

The structure of this paper is organized in the following folds: The Three-Diode Model (TDM) for PV cells with its relevant equations is introduced in section 2. Section 3 overviews the MTO-CL algorithm. Section 4 details the implementation setup for the ST40 and SM55 solar panels. The computational aspect of the algorithm is examined in Section 5, while the results analysis is elaborated on in Section 6. In section 7, we discuss Limitations of the proposed Methodology and future research. Finally, conclusion remarks including future work are highlighted in the last section.

## 2. PV modeling

PV modeling is a crucial element facilitating the estimation of its power and anticipating its operation. Therefore, this section will focus on the mathematical foundation of the three-diode model (TDM) in photovoltaic solar modules and cells, to which the MTO-CL algorithm will applied to estimate the PV parameters.

### 2.1 TDM and PV module modeling

**Fig 1:**
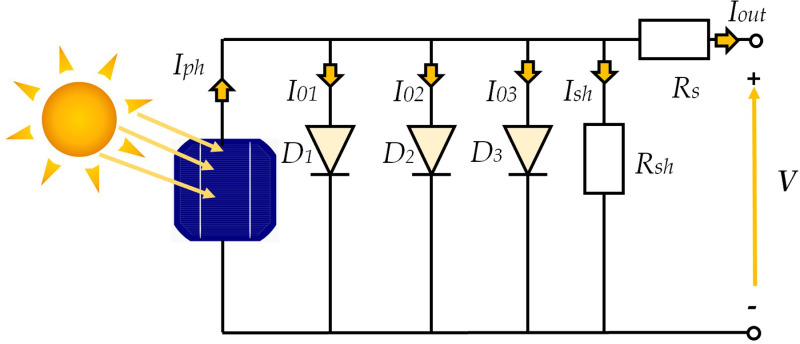
TDM equivalent circuit.


The corresponding solar PV-three-diode model (TDM) can be decomposed into a current source (Iph) parallelly connected to a resistor (Rsh) and three diodes with a further series connection with another resistor (Rs). The overall TDM model is described in Fig 1. The current Iph is distributed between the parallel diodes (I01, I02, I03) and resistor (Ish), resulting in the current generated by the photovoltaic unit (Iout), as demonstrated in Eq (1).

$$I_{out}=I_{ph}-\sum_{i=1}^{p}I_{oi}-\frac{V+I_{out}R_{s}}{R_{sh}}=I_{ph}-\sum_{i=1}^{p}I_{sti}(e^{q(\frac{V+I_{out}R_{s}}{n_{i}KT})}-1)-\frac{V+I_{out}R_{s}}{R_{sh}}$$ (1)

The symbol p represents the total of all paralleled diodes, specifically p = 3. V denotes the output voltage, while Ioi represents the current passing via diode i as presented in Eq (2).


$$\begin{array}{l} I_{oi}=I_{sti}(e^{q(\frac{V+I_{out}R_{s}}{n_{i}KT})}-1)=I_{ph}-I_{st3}(e^{q(\frac{V+I_{out}R_{s}}{n3KT})}-1)-\frac{V+I_{out}R_{s}}{R_{sh}}-I_{st2}(e^{q(\frac{V+I_{out}R_{s}}{n2KT})}-1)-\frac{V+I_{out}R_{s}}{R_{sh}}\\ -I_{st1}(e^{q(\frac{V+I_{out}R_{s}}{n1KT})}-1)-\frac{V+I_{out}R_{s}}{R_{sh}} \end{array}$$ (2)

The symbol Isti represents the saturated current, while q represents a one electron’s charge (1.602 ×10−19 C). K corresponds to Boltzmann’s constant, ni represents a diode’s ideality factor, and T denotes temperature (Kelvin).

**Fig 2:**
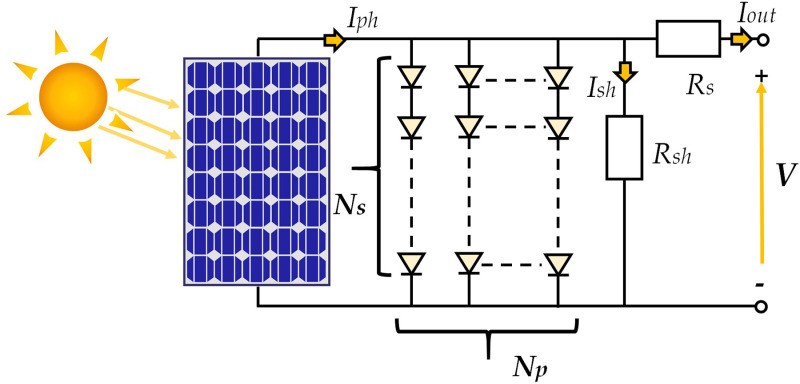
Photovoltaic Module.


In TDM-based PV module illustrated in Fig 2, comprising Ns× Np cells that are of parallel (and/or) series connection, the output current (Iout) from the solar cells can be demonstrated as in Eq (3):

$$I_{out}=I_{ph}-\sum_{i=1}^{p}I_{sti}(e^{q(\frac{V/N_{s}+(I_{out}R_{s})/N_{p}}{n_{i}KT})}-1)-\frac{V/N_{s}+(I_{out}R_{s})/N_{p}}{R_{sh}}$$ (3)

### 2.2 Objective function

The main focus of this endeavor is to minimize the gap among the generated current from the solar PV model and the actual measurement of current from solar PV cell. This can be attained through setting the objective function of the targeted problem as a minimization of the root mean square error (RMSE) among both values as demonstrated in Eq (4). Such a setting would ensure a fine tuning of the parameters of the solar PV model and achieving the best possible fit with relevance to the measured data. In this context, the Mother Tree Optimization with climate change (MTO-CL) algorithm is utilized to estimate the solar PV system’s parameters. Derived from the MTO algorithm, MTO-CL boosts the efficiency of the former algorithm by incorporating periodic climate change events. These events influence the algorithm’s behavior in the following critical phases: 1) elimination, and 2) distortion. During the first phase (elimination), solutions of the lowest fitness are substituted by new random solutions, comprising 20% of the overall population. In the second phase (distortion), remaining 80% of the population experiences slight positional variations. The MTO-CL algorithm adeptly balances (exploration & exploitation) in the parameters of the solar PV system, ensuring effective identification of the parameters’ optimal values that minimize RMSE. This will ensure improving the modeling accuracy and performance.


$$F_{\cos t}=\sqrt{\frac{1}{M}(\sum_{j=1}^{M}{\left|I_{es}-I_{mes}\right|}^{2})}=\sqrt{\frac{1}{M}(\sum_{j=1}^{M}{\left|I_{ph}-\sum_{i=1}^{p}I_{sti}(e^{q(\frac{V/N_{s}+(I_{out}R_{s})/N_{p}}{n_{i}KT})}-1)-\frac{V/N_{s}+(I_{out}R_{s})/N_{p}}{R_{sh}}-I_{mes}\right|}^{2})}$$ (4)

where Ies and Imes represent the calculated and measured currents, respectively. M represents an overall number of current data points.

## 3. MTO-CL algorithm

### 3.1 Biologic context

The MTO algorithm is inspired by a mutualistic link among Douglas fir trees and mycorrhizal fungi networks [47]. It is driven by three factors: kin recognition (Krs), kin selection (Ks), and the mycorrhizal fungi network (MFN). Krs affects tree growth as per the closeness to kin/non-kin. Ks helps gene propagation based on fitness improvement. MFN helps nutrient transfer among plants and thus, it supports their growth. Douglas fir trees provide carbohydrates while fungi provides nitrogen and phosphorus. MFNs enhance tree fitness via indirect protection against root pathogens. MTO algorithm systematically replaces least healthy trees to counter climate change impact [47].

### 3.2 Topology of fixed descent

**Fig 3:**
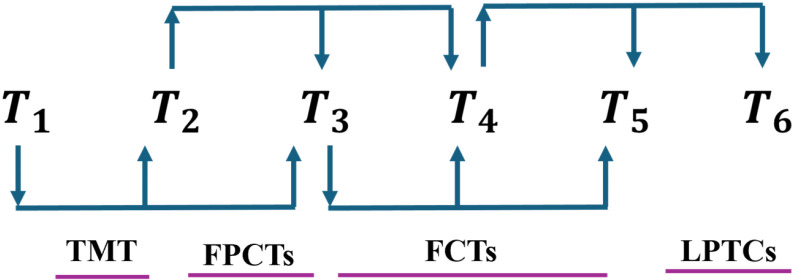
Variations among population subgroups and their nutrient uptake patterns.


MTO algorithm’s population can be decomposed into- 1) Top Mother Tree (TMT), 2) Partially Connected Trees (PCTs), and 3) Fully Connected Trees (FCTs). It is influenced by active food sources of size NT. TMT randomly receives nutrients, and PCTs and FCTs have agents of sizes NPCTs and NFCTs, respectively (See Eq 5). PCTs are further categorized into FPCTs and LPCTs, with NFPCTs and NLPCTs agents, respectively. FPCTs include (NT/2 - 2) agents from the second to the (NT/2 - 1)th rank, while LPCTs have (NT/2 - 2) agents from the second to the last rank. Fig 3 shows population division during nutrient allocation [47]. Individuals in MTO adjust locations as per AFS nutrients. TMT as a top-ranked agent would update position randomly without nutrient exchange. Agents ranked 1 to NT/2 + 1 nourish the next NOS = NT/2 - 1 trees. NFPCTs obtain nutrients from AFSs below NOS. On the other hand, NFCTs get nutrients from higher-ranked AFSs. NLPCTs get nutrients from AFSs below NOS, starting from NOS locations under their tree [47].

$$\left.\begin{array}{l} N_{PCTs}=N_{T}-4\\ N_{PCTs}=3\\ N_{T}=N_{PCTs}+1+N_{FTs} \end{array}\right\}$$ (5)

### 3.3. MTO-CL

TMT runs an exploitation phase with two levels. With a root step size of δ and, TMT assesses a new location randomly using Eqs (6) and (7), comparing nutrient intake for potential updates. If a higher nutrient count is found, the algorithm moves to the next level with a reduced step size Δ in (Eq 8).


$$P_{1}(x_{k}+1)=\delta R(d)+P_{1}(x_{k})$$ (6)

R(d)=2(round(rand(d,1))−1)rand(d,1) (7)

$$P_{1}(x_{k}+1)=\Delta R(d)+P_{1}(x_{k})$$ (8)

Where, R(d) denote a random vector that has been selected based on preliminary experiments. FPCTs’ positions are adjusted using Eq (9). If the new location has higher number of nutrients, it remains unchanged. Else, it would undergo adjustment by moving in a random direction, as specified in Eq (10), Pn(xk) is the actual location of a member within the range of (2, NT2 - 1), Pj(xk) reflects the actual location of a potential solution with a higher nutrient count, and Pn(xk+1) is the upgraded location of such a member as an additional condition when a member utilizes a defense mechanism as further described in [47].


$$P_{1}(x_{k}+1)=P_{1}(x_{k})+\sum_{j=1}^{m-1}\frac{1}{n-j+1}(P_{j}(x_{k})-P_{n}(x_{k}))$$ (9)

$$P_{n}(x_{k}+1)=\phi R(d)+P_{n}(x_{k})$$ (10)

where R denotes a random vector, and Φ indicates the small deviation from present position. When it comes to FCTs, the locations are adjusted as in Eq (11). LPCTs locations with candidates with the lowest nutrient levels undergo updates as in Eq (12) [47].


$$P_{n}(x_{k}+1)=P_{n}(x_{k})+\sum_{j=n-N_{m}}^{m-1}\frac{1}{n-j+1}(P_{j}(x_{k})-P_{n}(x_{k}))$$ (11)

$$P_{n}(x_{k}+1)=P_{n}(x_{k})+\sum_{j=n-N_{m}}^{N_{T}-N_{m}}\frac{1}{n-j+1}(P_{j}(x_{k})-P_{n}(x_{k}))$$ (12)

**Fig 4:**
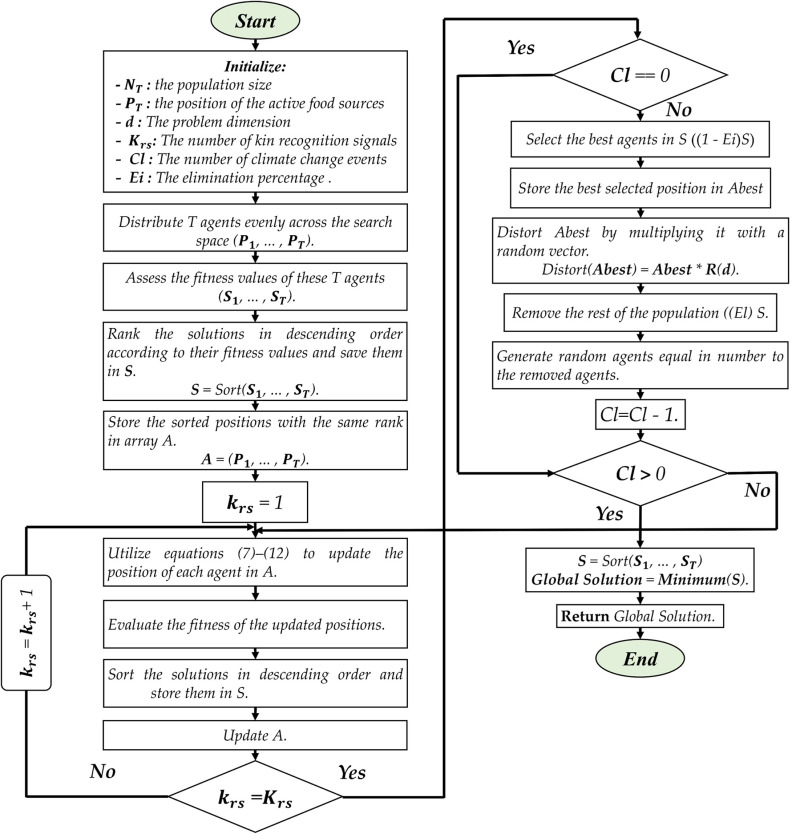
MTO algorithm.

MTO-CL presents a climate change event in two phases [47]: 1) elimination corresponding (Ei) to 20% of the population, removing low-fitness solutions and replacing them randomly, and 2) distortion corresponding to 80% of the population, introducing slight deviations in the locations of such population and consequently, promoting exploration and adaptation. MTO-CL follows cooperative behavior. In this case, exploitation is enhanced, and diversity is maintained. The climate change event, including Eq (10), simulates environmental changes. This makes MTO-CL robust and adaptable to dynamic optimization problems. Further elaboration on the MTO-CL algorithm is described in Fig 4.

The MTO-CL algorithm begins by initializing the population of agents and evaluating their fitness values. The agents are distributed uniformly over the search space, and their positions are sorted in decreasing rank of suitability. In each iteration corresponding to a kin recognition signal, the positions of the agents are updated using specific equations that simulate kin recognition and selection processes. After updating the positions, the fitness of the new positions is evaluated, and the solutions are sorted again based on their fitness values. This iterative update process helps refine the population and explore the search space effectively.

If a climate change event is enabled, the algorithm enters a specific phase. The best-performing agents are selected, and their positions are distorted by introducing small deviations. The rest of the population is removed, and random agents are generated to replace them. This climate change event simulates environmental changes and enhances the algorithm’s ability to adapt and find better solutions.

The algorithm continues to iterate and apply kin recognition updates until the specified number of climate change events is reached. At the end of the loop, the positions and fitness values are sorted again, and the global solution is determined as the agent with the minimum fitness value. This global solution represents the best solution found by the algorithm and is returned as the output.

### 3.4. Proposed algorithm

In this work, we apply the MTO-CL algorithm to optimize the solar PV TDM model. The optimization process aims to find the optimal solutions for the cost function defined in Eq (4). The size of the population of the algorithm is 40 with 2500 as the largest number of iterations.

**Fig 5:**
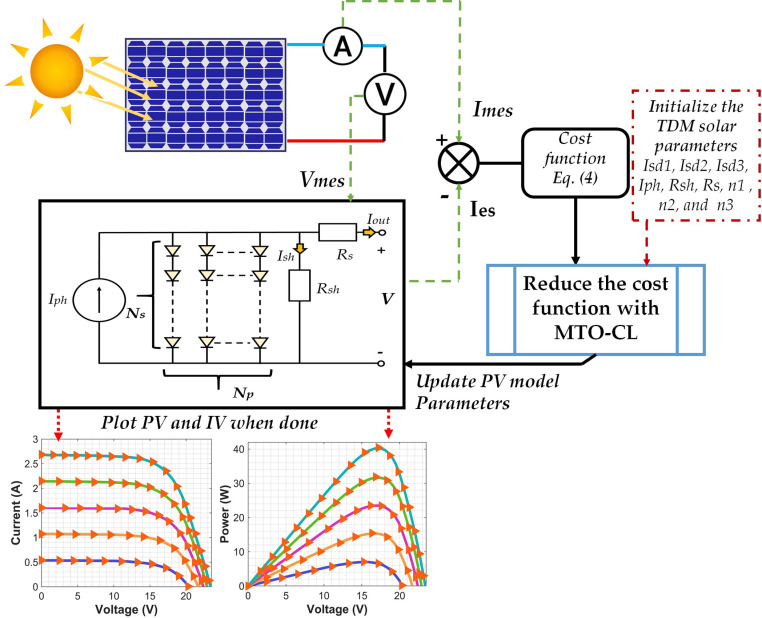
MTO-CL work flow.

The optimization procedure is summarized in Fig 5. The procedure’s initial step is to configure the PV model, and thus providing the input for computing the model’s parameters. The current and voltage are then read and utilized as model’s inputs.

Once the PV model and input metrics are in place, the MTO-CL algorithm moves on to locate the strongest candidate solution. This phase focuses on minimizing the cost function, defined in Eq (4), which serves as a performance assessment merit considering this model. The target is finding the model’s parameters of best alignment with the voltage and current metrics. Through an iterative reduction of the cost function, the MTO-CL algorithm is capable of finding the most promising solutions.

Finally, the MTO-CL algorithm outputs the optimal solution, which represents the closest correspondence between the evaluated and observed data. These optimized parameters provide the optimal configuration for the TDM system based on the specific cost function and performance criteria.

## 4. Setup requirements

The framework in Fig 5 is deployed to evaluate the parameters of the solar PV system. A comparative analysis is conducted in relevance to other benchmark optimization algorithms including Equilibrium Optimizer (EO) [48], Grey Wolf Optimizer (GWO) [49], Runge-Kutta Optimizer (RUN) [50], Slime Mould Algorithm (SMA) [51], Whale Optimization Algorithm (WOA) [52], Gradient-Based Optimizer (GBO) [53], and Gorilla Troops Optimizer (GTO) [54]. The evaluation is conducted for Siemens ST40 and Siemens SM55 PV modules [55].

The MTO-CL algorithm is run to find the PV solar system parameters as in Table 1 while considering the settings in Table 2. It is important to highlight that all the compared optimizers were initialized with identical configurations. Such a consideration will ensure a fair and consistent comparison.

**Table 1:** Datasheet parameters.

PV type	Solar module ST40	Solar module SM55
Maximum power rating P_max_ (W)	40	55
Rated current I_MPP_ (A)	2.41	3.15
Rated voltage V_MPP_ (V)	16.6	17.4
Short circuit current I_SC_ (A)	2.59	3.45
Open circuit voltage V_OC_ (W)	22.2	21.7

**Table 2:** MTO-CL parameters.

PV type	Solar module ST40	Solar module SM55
Population number (NT)	40	40
Number of maximum iterations	2500	2500
number of Decision variables (d)	9	9

In this study, we employed the heuristic algorithm as described in the original paper [47], utilizing the same fixed parameters to ensure consistency with previous research. These parameters have been optimized for similar contexts and are well-established in the field, providing a reliable basis for our estimation process. We selected the Population Number (NT) and Maximum Iterations based on commonly accepted practices to strike an optimal balance between accuracy and computational efficiency. While these settings are intended to offer a good tradeoff, we acknowledge that they could influence the convergence behavior and accuracy of the results.

## 5. Findings and discussion

**Fig 6:**
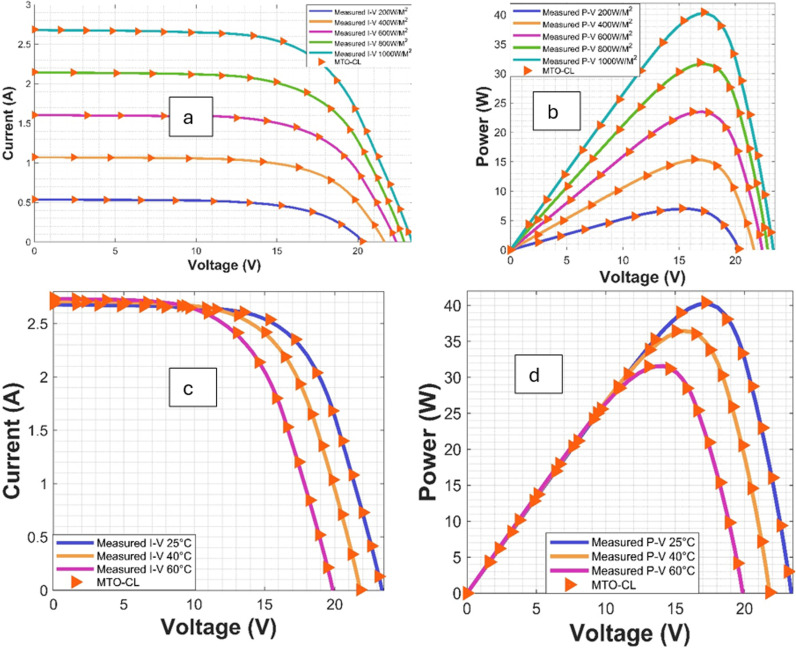
I-V and P-V Curves for ST40 Module: (a, b) Varied Irradiations, (c, d) Varied Temperatures.

**Fig 7:**
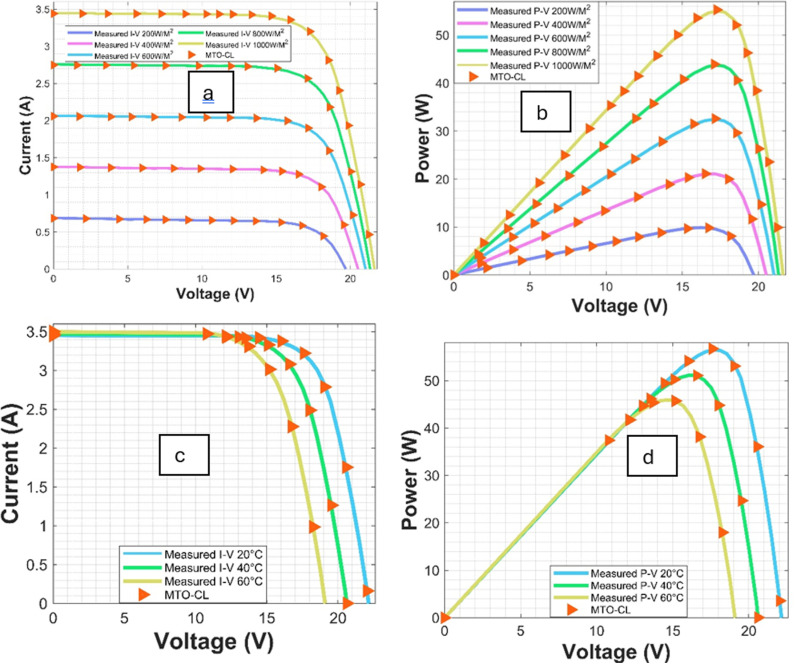
I-V and P-V Curves for SM55 Module: (a, b) Varied Irradiations, (c, d) Varied Temperatures.

The main goal of this work is to estimate the parameters essential for the TDM (Three-Diode Model). These parameters include Ist1, Ist2, Ist3, Iph, Rsh, Rs, n1, n2, and n3, which play a vibrant role in accurately modeling the solar modules. To assess the efficiency of the different optimization algorithms, the Power-Voltage (P-V) and Current-Voltage (C-V) characteristic curves of the solar PV module are analyzed under various irradiance and temperature levels, as diplayed in Figs 6 and 7, respectively.

**Fig 8:**
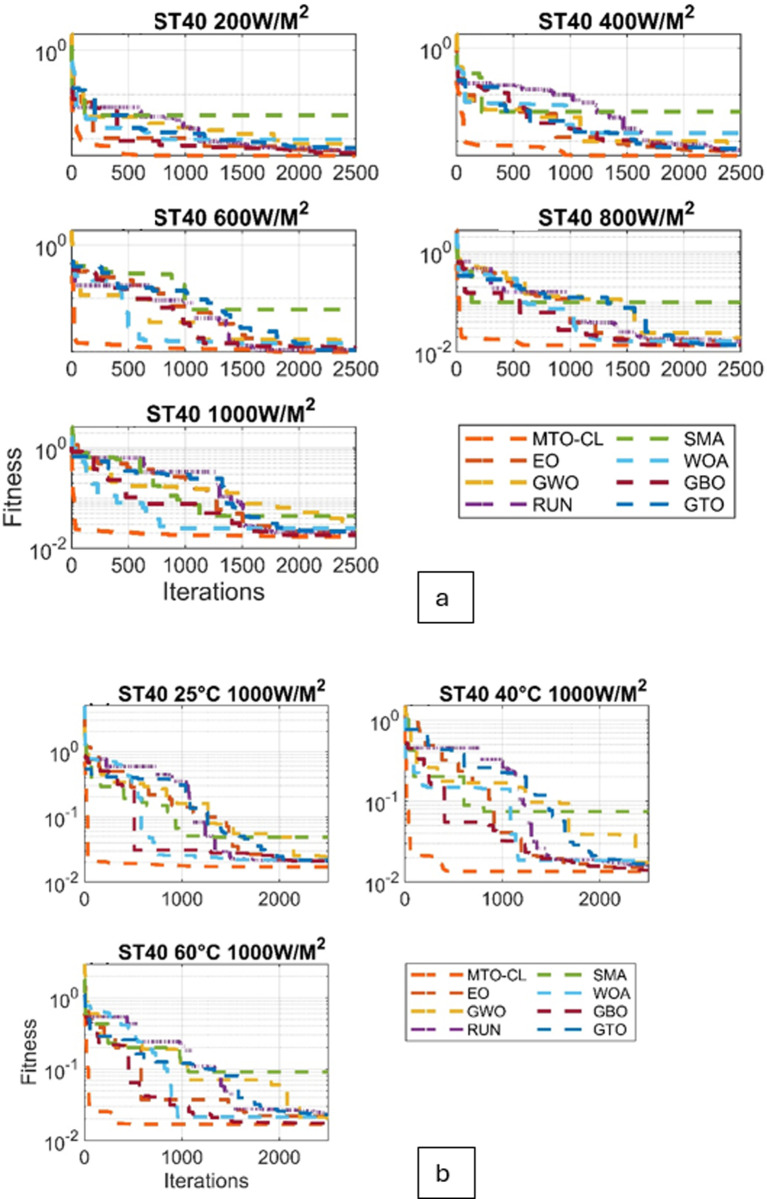
Fitness curves for ST40 Module: (a) Varied Irradiations, (b) Varied Temperatures.

**Fig 9:**
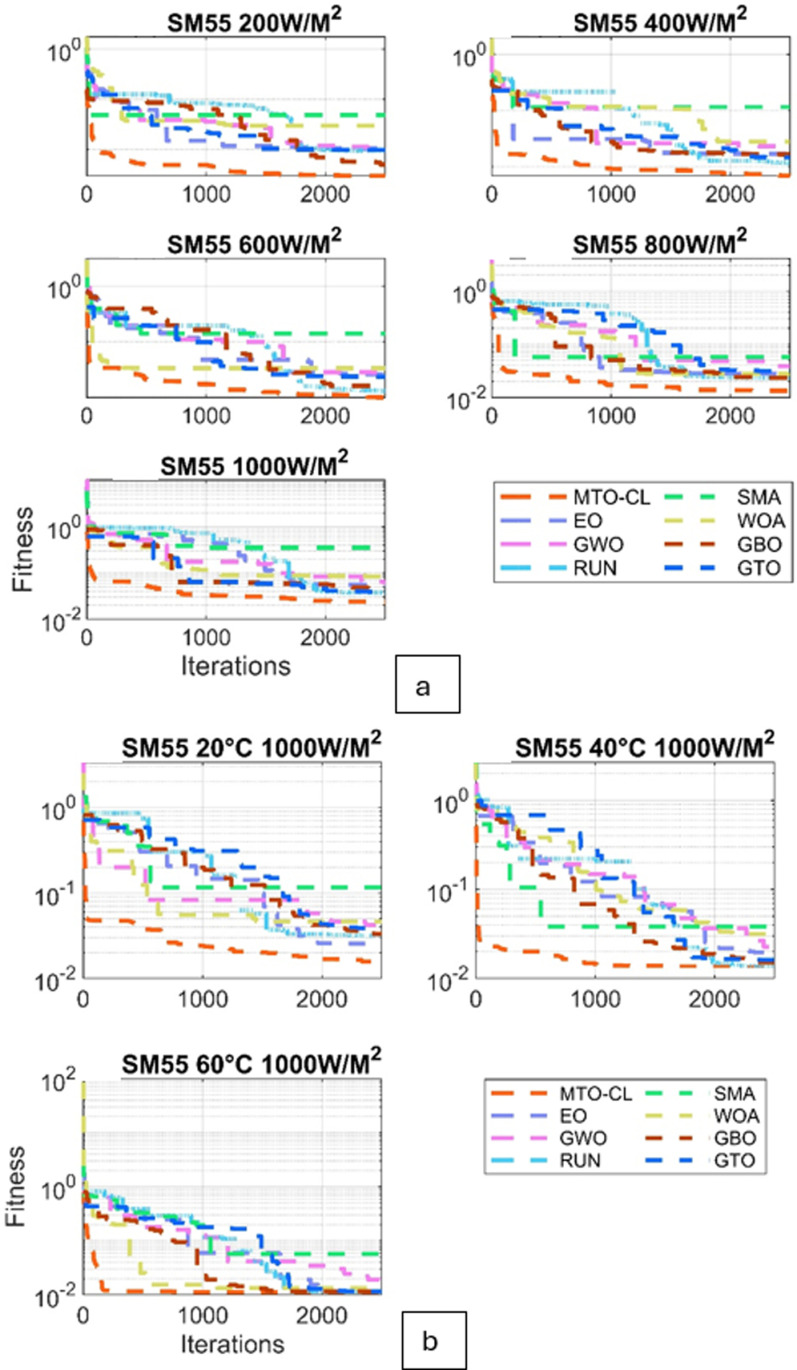
Fitness curves for SM55 Module: (a) Varied Irradiations, (b) Varied Temperatures.

**Fig 10:**
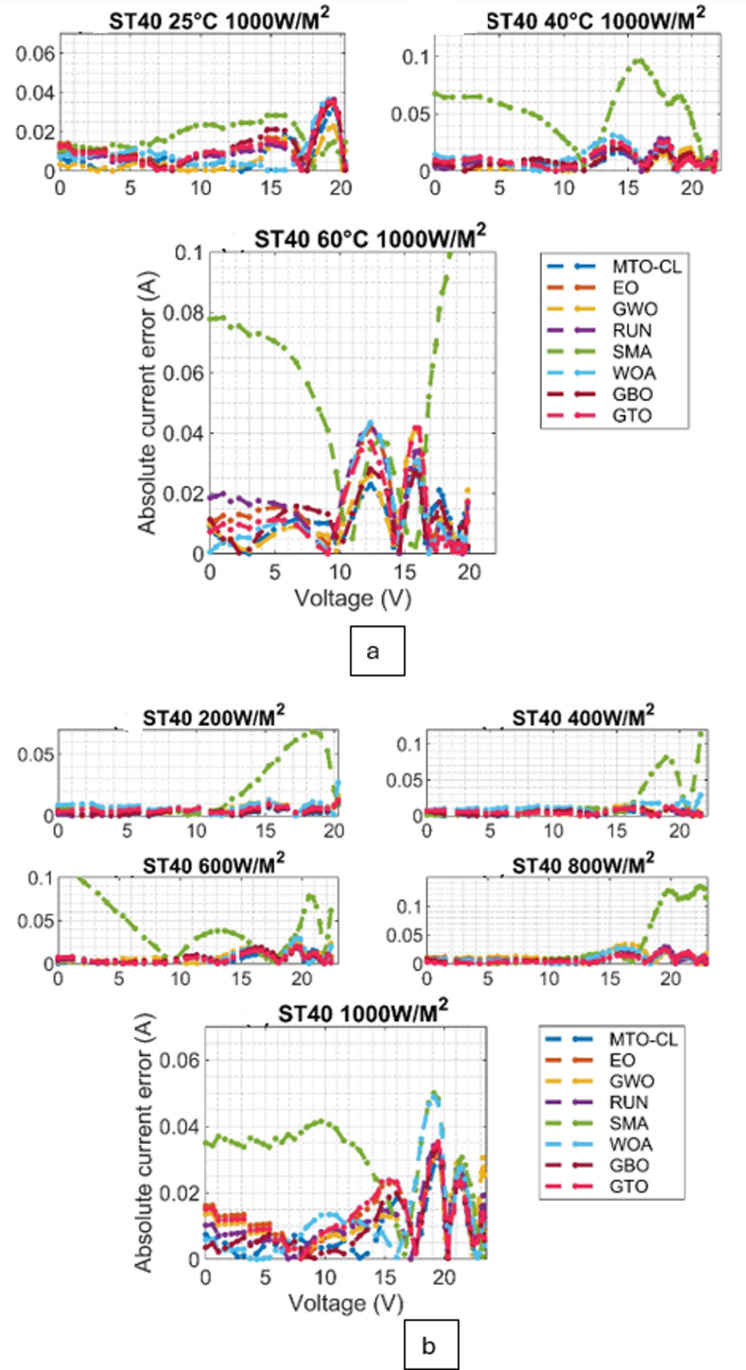
Absolute current error curves for ST40 Module: (a) Varied Irradiations, (b) Varied Temperatures.

**Fig 11:**
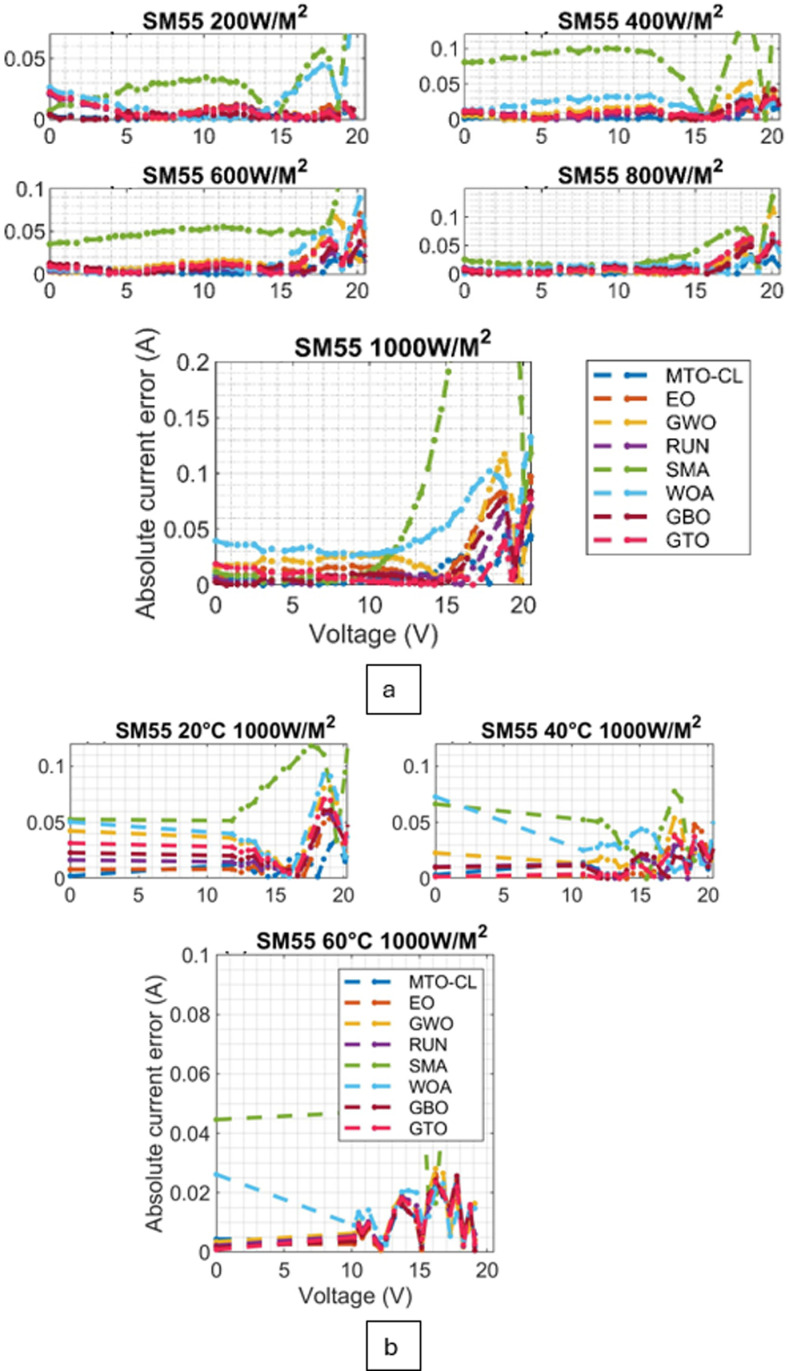
Absolute current error curves for SM55 Module: (a) Varied Irradiations, (b) Varied Temperatures.

Moreover, the convergence behavior of the various algorithms are examined through the converging plots shown in Figs 8 and 9. For evaluating the accuracy of estimating the parameter estimation, the absolute current errors are computed and demonstrated in Figs 10 and 11. Effective insights on the performance of the algorithms in recovering the desired parameters.

The performance of various optimization algorithms is examined through predictive performance indicators, with a focus on RMSE, Normalized Root Mean Square Error (NRMSE), and Mean Squared Error (MSE) for different solar PV types.


$$\text{MSE}=\frac{1}{m}{\sum_{k=1}^{m}\left(I_{es}(k)-{\text{I}}_{tr}(k)\right)}^{2}$$ (13)

$$\text{NRMSE}=\frac{RMSE}{I_{es,\max}-I_{es,\min}}$$ (14)

$$\text{RMSE}=\sqrt{\frac{1}{m}{\sum_{k=1}^{m}\left(I_{es}(k)-I_{tr}(k)\right)}^{2}}$$ (15)

The variables m, Ies, and Itr designate the point number, estimated and measured output currents, respectively.

**Fig 12:**
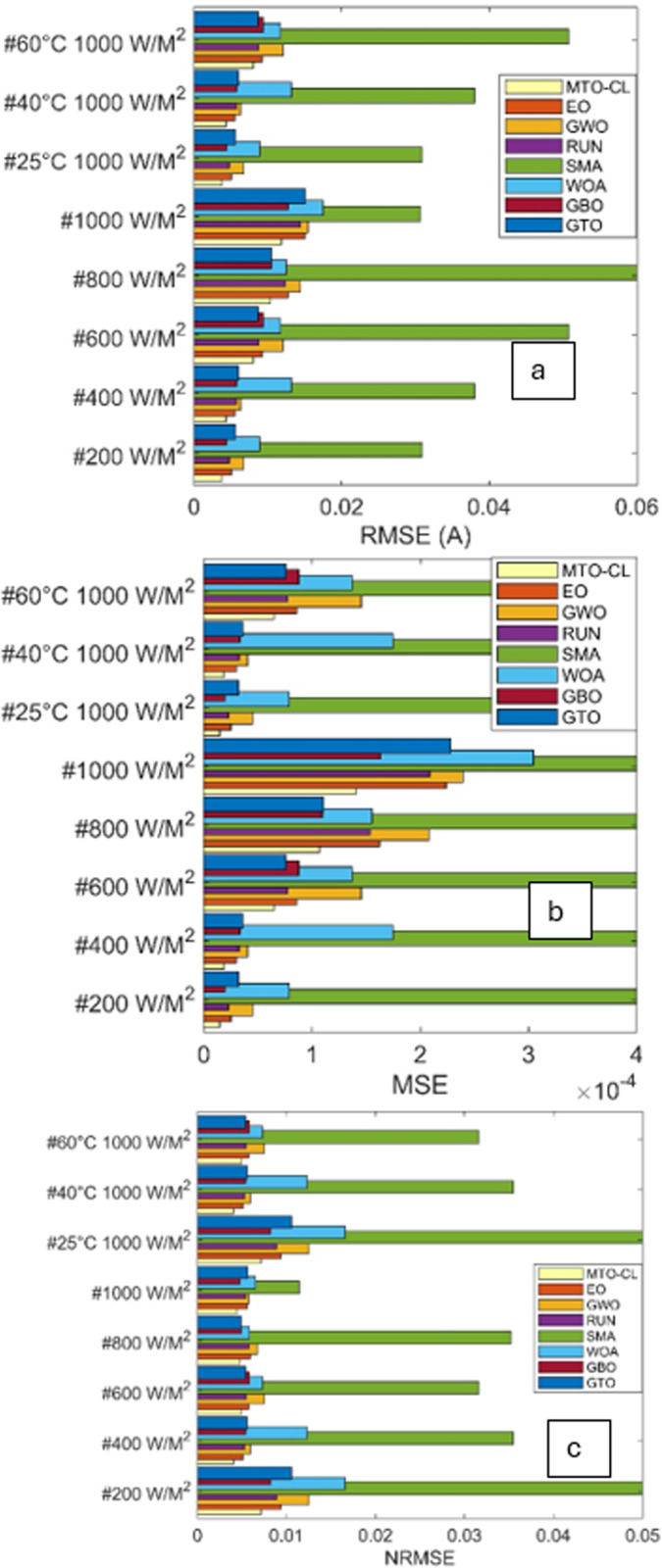
RMSE (a), MSE (b), and NRMSE (c) of all algorithms in ST40.

**Fig 13:**
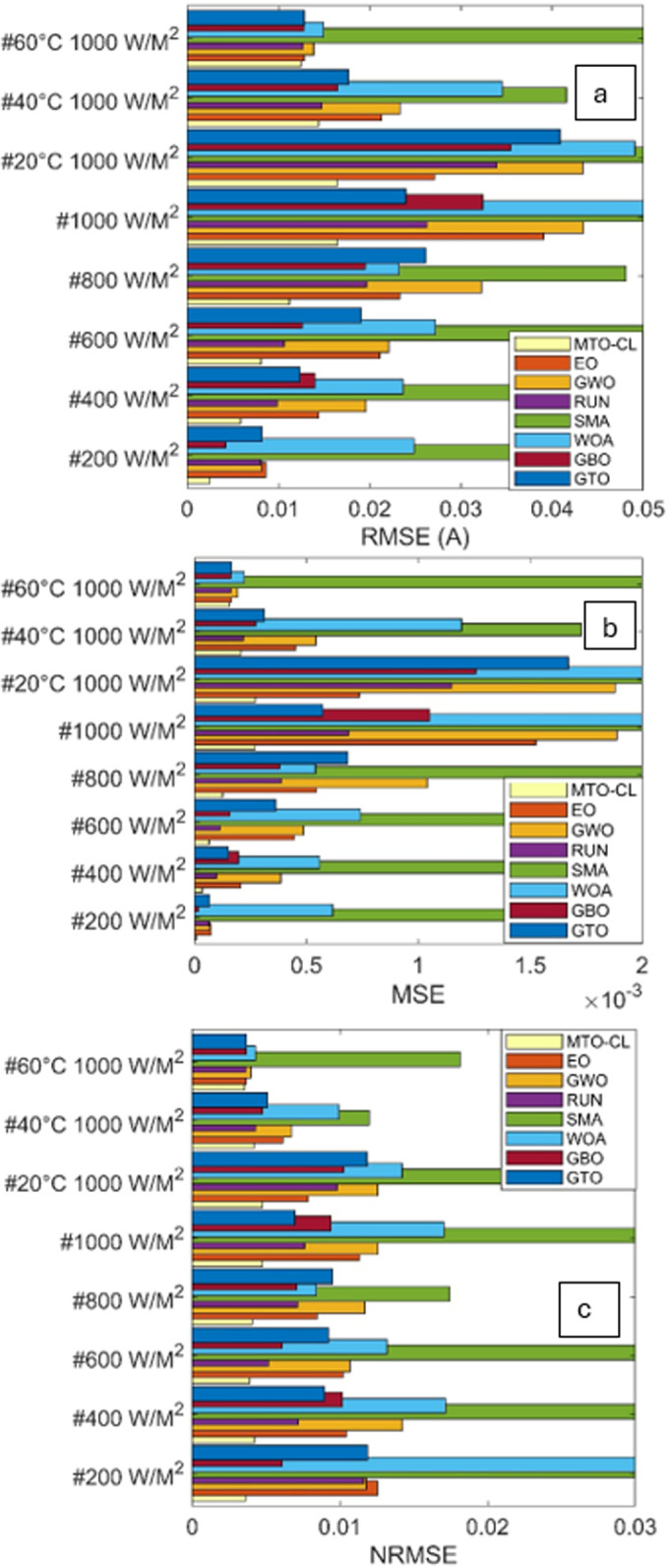
RMSE (a), MSE (b), and NRMSE (c) of all algorithms in SM55.

The results in Table 3, Figs 12 and 13 offer a comprehensive comparison. According to these results, the MTO-CL consistently demonstrates superior accuracy across all PV types (ST40 and SM55) under different irradiations and temperatures.

**Table 3:** Performance indicators predicting the performance of algorithms across various types of solar PV systems.

PV type	Error	Irradiance (W/M^2^)/ temperatures (°C)	MTO-CL	EO	GWO	RUN	SMA	WOA	GBO	GTO
**ST40 solar panel**	**RMSE (A)**	200(W/M^2^)/25 °C	0.0038	0.0050	0.0067	0.0048	0.0309	0.0089	0.0044	0.0057
		400(W/M^2^)/25 °C	0.0043	0.0055	0.0064	0.0057	0.0380	0.0132	0.0058	0.0060
		600(W/M^2^)/25 °C	0.0080	0.0093	0.0121	0.0088	0.0508	0.0117	0.0094	0.0087
		800(W/M^2^)/25 °C	0.0103	0.0127	0.0144	0.0124	0.0753	0.0125	0.0105	0.0105
		1000(W/M^2^)/25 °C	0.0119	0.0150	0.0155	0.0145	0.0306	0.0175	0.0128	0.0151
		1000(W/M^2^)/25 °C	0.0038	0.0050	0.0067	0.0048	0.0309	0.0089	0.0044	0.0057
		1000(W/M^2^)/40 °C	0.0043	0.0055	0.0064	0.0057	0.0380	0.0132	0.0058	0.0060
		1000(W/M^2^)/60 °C	0.0080	0.0093	0.0121	0.0088	0.0508	0.0117	0.0094	0.0087
	**MSE**	200(W/M^2^)/25 °C	1.5E-05	2.5E-05	4.5E-05	2.3E-05	9.6E-04	7.9E-05	1.9E-05	3.2E-05
		400(W/M^2^)/25 °C	1.9E-05	3.0E-05	4.0E-05	3.3E-05	1.4E-03	1.7E-04	3.3E-05	3.6E-05
		600(W/M^2^)/25 °C	6.5E-05	8.6E-05	1.5E-04	7.7E-05	2.6E-03	1.4E-04	8.8E-05	7.6E-05
		800(W/M^2^)/25 °C	1.1E-04	1.6E-04	2.1E-04	1.5E-04	5.7E-03	1.6E-04	1.1E-04	1.1E-04
		1000(W/M^2^)/25 °C	1.4E-04	2.2E-04	2.4E-04	2.1E-04	9.4E-04	3.0E-04	1.6E-04	2.3E-04
		1000(W/M^2^)/20 °C	1.5E-05	2.5E-05	4.5E-05	2.3E-05	9.6E-04	7.9E-05	1.9E-05	3.2E-05
		1000(W/M^2^)/40 °C	1.9E-05	3.0E-05	4.0E-05	3.3E-05	1.4E-03	1.7E-04	3.3E-05	3.6E-05
		1000(W/M^2^)/60 °C	6.5E-05	8.6E-05	1.5E-04	7.7E-05	2.6E-03	1.4E-04	8.8E-05	7.6E-05
	**NRMSE**	200(W/M^2^)/25 °C	0.0071	0.0093	0.0125	0.0089	0.0577	0.0166	0.0082	0.0105
		400(W/M^2^)/25 °C	0.0040	0.0051	0.0059	0.0053	0.0354	0.0123	0.0054	0.0056
		600(W/M^2^)/25 °C	0.0050	0.0058	0.0075	0.0055	0.0316	0.0073	0.0058	0.0054
		800(W/M^2^)/25 °C	0.0048	0.0060	0.0067	0.0058	0.0352	0.0058	0.0049	0.0049
		1000(W/M^2^)/25 °C	0.0044	0.0056	0.0058	0.0054	0.0114	0.0065	0.0048	0.0056
		1000(W/M^2^)/20 °C	0.0071	0.0093	0.0125	0.0089	0.0577	0.0166	0.0082	0.0105
		1000(W/M^2^)/40 °C	0.0040	0.0051	0.0059	0.0053	0.0354	0.0123	0.0054	0.0056
		1000(W/M^2^)/60 °C	0.0050	0.0058	0.0075	0.0055	0.0316	0.0073	0.0058	0.0054
**SM55 solar panel**	**RMSE (A)**	200(W/M^2^)/25 °C	0.0025	0.0087	0.0081	0.0080	0.0405	0.0248	0.0042	0.0082
		400(W/M^2^)/25 °C	0.0058	0.0144	0.0196	0.0099	0.0974	0.0236	0.0140	0.0123
		600(W/M^2^)/25 °C	0.0081	0.0211	0.0221	0.0107	0.1145	0.0272	0.0126	0.0191
		800(W/M^2^)/25 °C	0.0112	0.0233	0.0322	0.0196	0.0481	0.0232	0.0195	0.0262
		1000(W/M^2^)/25 °C	0.0165	0.0391	0.0434	0.0262	0.2473	0.0588	0.0324	0.0240
		1000(W/M^2^)/25 °C	0.0165	0.0271	0.0434	0.0339	0.1242	0.0491	0.0355	0.0409
		1000(W/M^2^)/40 °C	0.0145	0.0213	0.0233	0.0148	0.0416	0.0345	0.0165	0.0177
		1000(W/M^2^)/60 °C	0.0125	0.0128	0.0139	0.0127	0.0635	0.0149	0.0128	0.0128
	**MSE**	200(W/M^2^)/25 °C	6.2E-06	7.5E-05	6.6E-05	6.4E-05	1.6E-03	6.2E-04	1.8E-05	6.7E-05
		400(W/M^2^)/25 °C	3.4E-05	2.1E-04	3.9E-04	9.8E-05	9.5E-03	5.6E-04	2.0E-04	1.5E-04
		600(W/M^2^)/25 °C	6.5E-05	4.5E-04	4.9E-04	1.1E-04	1.3E-02	7.4E-04	1.6E-04	3.6E-04
		800(W/M^2^)/25 °C	1.3E-04	5.4E-04	1.0E-03	3.9E-04	2.3E-03	5.4E-04	3.8E-04	6.9E-04
		1000(W/M^2^)/25 °C	2.7E-04	1.5E-03	1.9E-03	6.9E-04	6.1E-02	3.5E-03	1.0E-03	5.7E-04
		1000(W/M^2^)/20 °C	2.7E-04	7.4E-04	1.9E-03	1.1E-03	1.5E-02	2.4E-03	1.3E-03	1.7E-03
		1000(W/M^2^)/40 °C	2.1E-04	4.5E-04	5.4E-04	2.2E-04	1.7E-03	1.2E-03	2.7E-04	3.1E-04
		1000(W/M^2^)/60 °C	1.6E-04	1.7E-04	1.9E-04	1.6E-04	4.0E-03	2.2E-04	1.6E-04	1.6E-04
	**NRMSE**	200(W/M^2^)/25 °C	0.0036	0.0126	0.0118	0.0116	0.0587	0.0360	0.0061	0.0119
		400(W/M^2^)/25 °C	0.0042	0.0105	0.0143	0.0072	0.0707	0.0172	0.0102	0.0090
		600(W/M^2^)/25 °C	0.0039	0.0102	0.0107	0.0052	0.0554	0.0132	0.0061	0.0092
		800(W/M^2^)/25 °C	0.0041	0.0085	0.0117	0.0071	0.0174	0.0084	0.0071	0.0095
		1000(W/M^2^)/25 °C	0.0048	0.0113	0.0126	0.0076	0.0717	0.0171	0.0094	0.0069
		1000(W/M^2^)/20 °C	0.0048	0.0079	0.0126	0.0098	0.0360	0.0142	0.0103	0.0118
		1000(W/M^2^)/40 °C	0.0042	0.0061	0.0067	0.0043	0.0120	0.0099	0.0048	0.0051
		1000(W/M^2^)/60 °C	0.0036	0.0037	0.0040	0.0036	0.0182	0.0043	0.0037	0.0037

### ST40 solar panel

Regarding RMSE, the MTO-CL algorithm demonstrated remarkable accuracy across varying irradiance levels and temperatures. For instance, at 200(W/M2)/25 °C, MTO-CL has achieved the lowest RMSE of 0.0038, demonstrating its superior performance. This trend continued across different conditions, with MTO-CL consistently outperforming other algorithms, achieving the lowest RMSE of 0.0119 at 1000(W/M2)/25 °C.

Moving on to MSE, MTO-CL maintained its excellence, which is particularly clear at 400(W/M2)/25 °C, where it exhibited the lowest MSE of 1.9 × 10−05. This pattern continues at 1000(W/M2)/25 °C, where MTO-CL consistently has showen the lowest MSE of 1.4 × 10−04.

NRMSE comparisons further underscored the reliability of the MTO-CL algorithm. Across various conditions, the MTO-CL algorithm continues to display the lowest NRMSE values, which confirms its accuracy in predicting the performance of ST40 solar panels.

### SM55 solar panel

The evaluation of the SM55 solar panel has demonstrated a similar pattern of superiority in terms of the performance of the MTO-CL algorithm. At 200(W/M2)/25 °C, MTO-CL has achieved the lowest RMSE of 0.0025, which reflects its effectiveness. This trend continues at 1000(W/M2)/25 °C, where MTO-CL once again has demonstrated the lowest RMSE of 0.0165.

In terms of MSE, MTO-CL consistently outperformed other algorithms, with notable instances as at 600(W/M2)/25 °C, where it has exhibited the lowest MSE of 4.9 × 10−04. This trend continues at 1000(W/M2)/25 °C, where MTO-CL has displayed the lowest MSE of 2.7 × 10−04.

The NRMSE comparisons reaffirms the reliability of the MTO-CL algorithm for the SM55 solar panel as it consistently demonstrates the lowest NRMSE values across different conditions.

In nutshell, the MTO-CL algorithm has proven its efficiacy in accurately estimating the essential parameters to the Three-Diode Model in both ST40 and SM55 solar panels. The numerical results, in the form of RMSE, MSE, and NRMSE, reflects the superiority of the MTO-CL over other algorithms in the predictive modeling of the various solar PV systems.

Table 4 shows the performance of various algorithms across the different solar PV systems, focusing on critical parameters such as the maximum current, mean current, and power error (as defined in Eq (16)). It is important to note that a lower value of the error reflects a better algorithm’s performance. The evaluation encompasses different solar irradiance levels and temperatures, to verify the efficacy of the selected algorithm (MTO-CL) among other algorithms (EO, GWO, RUN, SMA, WOA, GBO, and GTO).

**Table 4:** Absolute maximum current, mean current, and power error for all evaluated algorithms.

PV type	Error	Irradiance (W/M^2^)/ temperatures (°C)	MTO-CL	EO	GWO	RUN	SMA	WOA	GBO	GTO
**ST40 solar panel**	**MAX (mA)**	200(W/M^2^)/25 °C	9.33	14.52	16.56	12.46	68.39	27.19	11.84	12.71
		400(W/M^2^)/25 °C	10.50	11.22	15.51	10.86	114.01	22.22	12.04	9.91
		600(W/M^2^)/25 °C	15.34	16.50	23.52	15.95	121.79	20.06	19.08	15.16
		800(W/M^2^)/25 °C	18.67	25.73	33.14	23.74	19.38	27.03	17.92	20.48
		1000(W/M^2^)/25 °C	23.11	24.84	29.05	27.93	41.55	27.68	23.01	23.75
		1000(W/M^2^)/25 °C	22.61	28.24	30.26	27.04	28.40	32.36	24.31	28.90
		1000(W/M^2^)/40 °C	16.70	22.91	21.11	20.47	96.20	31.35	19.95	25.58
		1000(W/M^2^)/60 °C	22.95	41.87	25.97	43.25	155.13	43.46	28.06	37.04
	**Mean (mA)**	200(W/M^2^)/25 °C	3.07	4.00	5.65	3.82	20.92	7.53	3.59	5.16
		400(W/M^2^)/25 °C	3.29	4.30	5.04	5.07	23.68	11.98	4.79	5.15
		600(W/M^2^)/25 °C	6.23	7.03	9.54	6.98	41.76	8.67	7.72	6.85
		800(W/M^2^)/25 °C	8.23	10.52	11.91	9.96	52.39	9.95	8.42	7.90
		1000(W/M^2^)/25 °C	9.24	12.97	12.79	11.70	27.85	12.35	9.84	12.82
		1000(W/M^2^)/20 °C	9.08	12.47	9.53	11.56	24.92	11.61	12.13	12.37
		1000(W/M^2^)/40 °C	8.82	9.50	8.74	9.27	49.63	11.66	9.31	10.44
		1000(W/M^2^)/60 °C	10.95	13.69	11.89	14.93	60.91	11.99	11.57	13.06
	**Power error (mW)**	200(W/M^2^)/25 °C	39.84	56.24	73.18	55.20	329.24	85.07	48.11	62.64
		400(W/M^2^)/25 °C	52.96	62.24	70.77	62.94	430.38	162.89	63.04	61.90
		600(W/M^2^)/25 °C	105.84	117.85	159.75	111.87	482.80	141.14	118.95	109.21
		800(W/M^2^)/25 °C	140.38	159.91	172.34	155.41	1057.91	160.59	141.80	142.53
		1000(W/M^2^)/25 °C	157.22	192.32	208.72	193.25	327.08	212.87	23.01	191.43
		1000(W/M^2^)/20 °C	155.01	193.99	180.91	193.20	424.69	188.64	187.18	198.98
		1000(W/M^2^)/40 °C	136.10	143.87	139.90	143.26	642.96	162.06	138.21	148.43
		1000(W/M^2^)/60 °C	150.51	161.44	163.82	164.99	789.70	156.47	149.22	163.76
**SM55 solar panel**	**MAX (mA)**	200(W/M^2^)/25 °C	6.04	12.15	10.33	11.11	166.15	100.55	13.19	11.98
		400(W/M^2^)/25 °C	15.13	42.42	38.39	26.20	265.69	33.55	41.96	30.67
		600(W/M^2^)/25 °C	24.42	70.29	26.71	35.75	379.87	89.04	37.51	60.80
		800(W/M^2^)/25 °C	28.33	63.27	114.98	55.63	135.51	66.74	56.47	69.12
		1000(W/M^2^)/25 °C	43.91	97.13	68.11	70.49	748.58	132.38	83.87	77.25
		1000(W/M^2^)/25 °C	31.26	51.87	69.47	68.03	150.05	75.43	68.81	72.00
		1000(W/M^2^)/40 °C	25.47	48.06	24.87	26.45	66.40	72.86	37.36	37.25
		1000(W/M^2^)/60 °C	24.51	24.08	19.31	21.42	93.29	26.14	25.64	21.99
	**Mean (mA)**	200(W/M^2^)/25 °C	2.04	7.20	6.36	6.40	31.02	16.14	3.33	6.35
		400(W/M^2^)/25 °C	3.90	9.67	14.57	8.12	86.09	21.66	10.53	9.95
		600(W/M^2^)/25 °C	5.90	13.78	15.08	6.74	78.20	17.37	9.78	13.20
		800(W/M^2^)/25 °C	8.05	15.75	16.46	13.27	36.52	16.93	13.38	16.23
		1000(W/M^2^)/25 °C	11.85	27.94	33.74	17.11	160.64	50.89	20.91	15.34
		1000(W/M^2^)/20 °C	13.48	21.06	36.85	27.15	105.12	41.36	29.47	34.98
		1000(W/M^2^)/40 °C	11.21	14.88	19.75	11.59	35.65	30.58	13.04	12.70
		1000(W/M^2^)/60 °C	10.40	10.28	11.53	10.98	59.61	13.47	10.31	10.69
	**Power error (mW)**	200(W/M^2^)/25 °C	22.86	65.29	50.62	53.57	394.10	213.82	38.83	50.86
		400(W/M^2^)/25 °C	58.35	135.56	209.92	98.08	967.45	241.43	144.33	127.45
		600(W/M^2^)/25 °C	76.86	217.87	226.29	105.46	1154.84	286.76	133.21	202.28
		800(W/M^2^)/25 °C	113.78	254.42	294.72	212.85	547.59	251.34	212.40	274.63
		1000(W/M^2^)/25 °C	195.03	460.79	507.19	297.92	2926.90	751.95	83.87	244.06
		1000(W/M^2^)/20 °C	239.40	391.55	626.94	499.51	1885.44	705.44	529.59	620.08
		1000(W/M^2^)/40 °C	185.70	270.92	309.68	189.07	503.95	434.57	218.34	224.53
		1000(W/M^2^)/60 °C	155.45	156.64	171.36	163.43	838.86	179.81	156.74	160.90

### Maximum current (mA)

Examining the maximum current predictions across the ST40 and SM55 solar panels reveals variations among the algorithms. For the ST40 panel, MTO-CL consistently leads the pack, demonstrating a maximum current value ranging from 9.33 mA to 68.39 mA. In contrast, EO, GWO, and RUN display competitive yet slightly lower values. For the SM55 panel, MTO-CL’s dominance continues, with a maximum current value ranging from 6.04 mA to 166.15 mA. This substantial difference highlights that MTO-CL’s is superior in accurately predicting the peak current output for both solar panel types. The numerical results highlight that MTO-CL’s is reliable and effective in capturing the dynamic behavior of solar panels under varying conditions.

### Mean current (mA)

When it comes to the mean current predictions, MTO-CL once again stands out in performance for both solar panels. In the case of the ST40 panel, MTO-CL consistently provides mean current values ranging from 3.07 mA to 10.95 mA. This robust performance signifies MTO-CL’s ability to capture the average current behavior accurately. Other algorithms, while still being competitive, demonstrate a slightly higher spread in their predictions. For the SM55 panel, MTO-CL maintains its superiority with mean current values ranging from 2.04 mA to 11.85 mA. The narrower range and lower mean current error further underscore MTO-CL’s precision in estimating the average current. Such observation solidify the algorithm position as a reliable choice for the accurate modeling of the solar PV systems.

### Power error (mW)

The evaluation of power error values provides crucial insights into the algorithms’ ability to predict the overall power variations. Across both solar panel types, MTO-CL consistently exhibits lower power error values compared to other algorithms. For the ST40 panel, MTO-CL’s power error, described through Eq (16), ranges from 39.84 mW to 150.51 mW, which reflects the algorithm’s proficiency in capturing the power variations accurately. This trend continues for the SM55 panel, where MTO-CL’s power error ranges from 22.86 mW to 195.03 mW. The substantially lower power error underscores MTO-CL’s effectiveness in predicting the power output across diverse irradiance levels and temperature conditions. These numerical results substantiate MTO-CL as a reliable and precise algorithm for predicting the power output of the solar PV systems.

The MTO-CL algorithm consistently emerges as a favorable algorithm choice given its superiority in accurately predicting the critical parameters of the PV model across different scenarios. Its performance, as reflected in the maximum, mean, and power error values, underscores its efficacy in enhancing the precision of predictions for both ST40 and SM55 solar panels.


$$\text{P\_error}=\frac{1}{m}\sqrt{\sum_{i=1}^{m}\left|P_{meas}(i)-P_{est}(i)\right|}$$ (16)

$$\text{RMS\_Power}=\frac{1}{m}\sqrt{\sum_{i=1}^{m}\left(P_{meas}(i)-P_{est}(i\right){)}^{2}}$$ (17)

where Pmeas and Pest represent he actual and estimated powers, while m corresponds to the number of data points.

**Fig 14:**
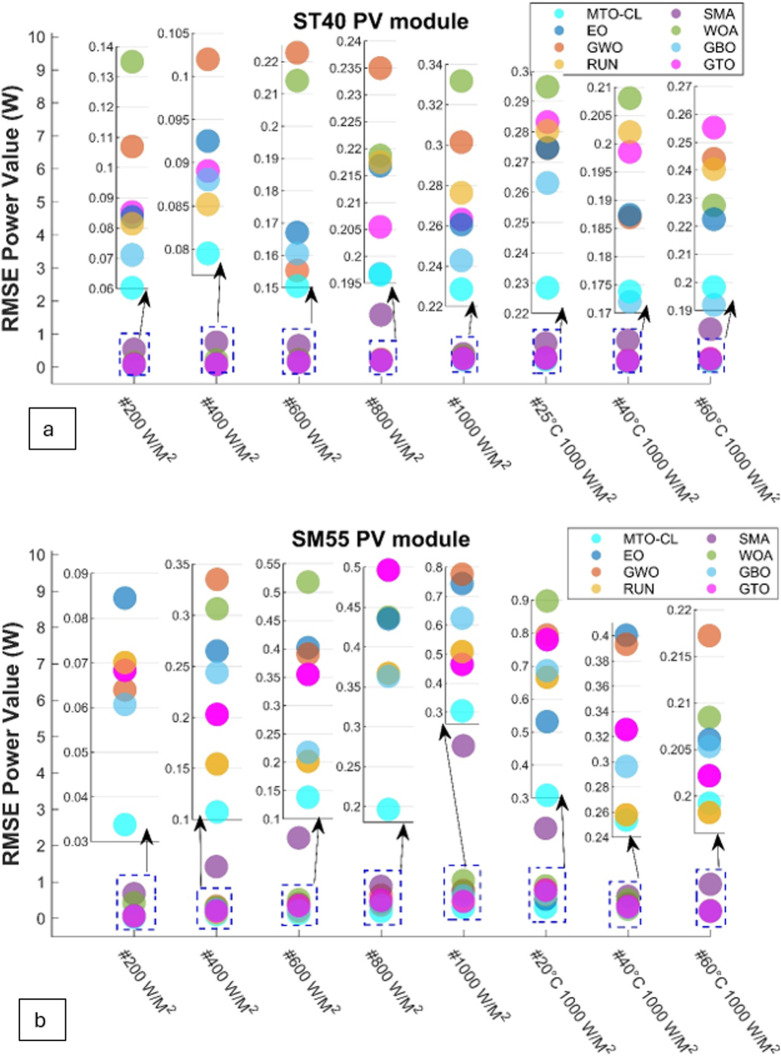
RMSE power of all algorithms: (a) ST40, (b) SM55 modules.

The RMSE power that is demonstrated by in Eq (17) is plotted in Fig 14. The plot validates the preeminence of the MTO-CL algorithm in precision, surpassing all benchmark algorithms across all tests by a significant margin.

## 6. Computation speed

**Fig 15:**
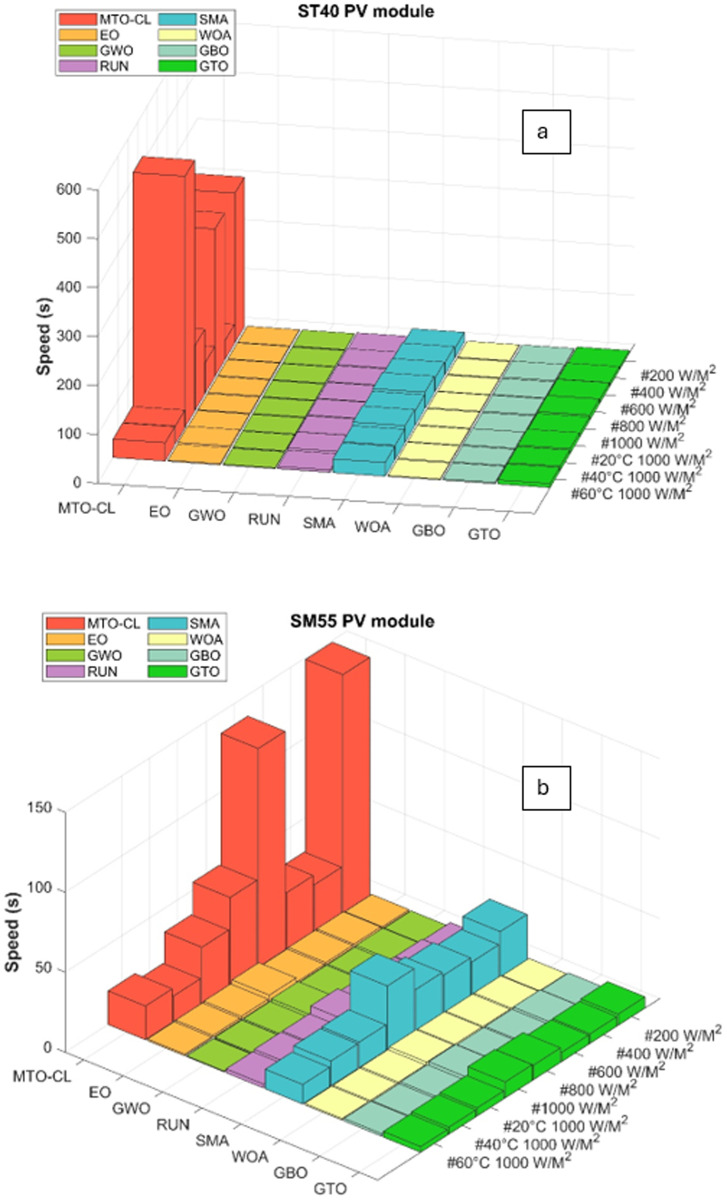
Computation speed of the algorithms: (a) ST40, (b) SM55 modules.

This section offers an assessment of the speed of the tackled optimization algorithms. For the implemented algorithms, the execution times of such algorithms are calculated. Table 5 and Fig 15 demonstrate the results. Table 5 displays the processing speed in seconds for every algorithm used for the four types of solar PV. The population size of all algorithms is 40 and the largest iteration rate is 2500.

**Table 5:** Processing speed in seconds.

PV type	Irradiance (W/M^2^)/ temperatures (°C)	MTO-CL	EO	GWO	RUN	SMA	WOA	GBO	GTO
**ST40 solar panel**	200(W/M^2^)/25 °C	305.75	1.05	0.93	2.98	19.09	1.41	1.44	4.87
	400(W/M^2^)/25 °C	38.81	1.72	1.01	5.22	24.49	1.45	3.25	6.93
	600(W/M^2^)/25 °C	301.58	2.18	1.64	5.27	27.41	1.40	2.14	8.15
	800(W/M^2^)/25 °C	62.49	2.14	1.52	5.61	33.30	2.10	2.67	9.71
	1000(W/M^2^)/25 °C	132.73	1.73	1.39	6.17	29.49	1.66	1.71	7.76
	1000(W/M^2^)/25 °C	513.46	1.54	1.64	6.58	34.12	2.02	3.34	9.99
	1000(W/M^2^)/40 °C	36.60	4.07	1.78	7.91	28.43	2.42	2.76	8.83
	1000(W/M^2^)/60 °C	37.49	2.09	1.32	4.82	27.89	1.60	2.61	8.13
**SM55 solar panel**	200(W/M^2^)/25 °C	148.96	1.22	0.99	3.75	28.41	1.36	2.37	6.96
	400(W/M^2^)/25 °C	29.02	1.75	1.56	4.62	20.80	1.87	3.14	5.95
	600(W/M^2^)/25 °C	35.17	1.53	0.97	4.84	23.88	1.82	2.39	5.78
	800(W/M^2^)/25 °C	136.74	1.09	1.04	5.29	26.15	2.01	2.32	9.10
	1000(W/M^2^)/25 °C	54.91	3.89	2.14	7.69	39.78	2.18	3.03	9.94
	1000(W/M^2^)/20 °C	35.14	1.94	0.98	3.39	15.99	1.06	1.72	5.01
	1000(W/M^2^)/40 °C	16.07	1.42	0.75	3.29	15.51	0.92	1.21	4.33
	1000(W/M^2^)/60 °C	21.06	1.00	0.51	2.41	12.19	0.93	1.58	3.46

A notable observation from the numerical results is that the MTO-CL algorithm demonstrates comparatively slower computation speed. For example, for the ST40 type, MTO-CL takes between 36 or 60 and 513 or 46 seconds, whereas EO and GWO take under 4 seconds each. However, as highlighted in the results presented in Tables 3 and 4, the MTO-CL stands out as the most accurate algorithm. This underscores the inherent tradeoff between accuracy and speed in the algorithmic modeling.

Specifically, for all PV types (ST40 and SM55 modules), MTO-CL consistently outperforms other algorithms in terms of accuracy, although at a slower computational pace.

It is important to note that the decision to prioritize accuracy over speed is a deliberate choice, recognizing the significance of precise modeling in the field of photovoltaics. While MTO-CL may have a longer computation time, the reliability of its results positions it as a superior choice for applications where accuracy is paramount.

## 7. Limitations of the proposed methodology and future research

The enhanced Mother Tree Optimization with Constraint Learning (MTO-CL) methodology presents notable advantages in terms of accuracy, particularly in the context of parameter extraction for three-diode photovoltaic (PV) systems. However, several limitations and areas for improvement have been identified:

- **Computational Speed:** One of the primary limitations of the MTO-CL algorithm is its slower computation speed compared to other optimization algorithms like EO (Evolutionary Optimization) and GWO (Grey Wolf Optimization). As shown in the results, MTO-CL takes significantly longer to process (between 36 to 513 seconds) compared to the sub-4-second execution times of EO and GWO. This slower pace can be a critical drawback, especially in applications where time efficiency is essential.
- **Tradeoff Between Accuracy and Speed:** Although MTO-CL excels in accuracy, this advantage comes at the cost of increased computational time. This inherent tradeoff between accuracy and speed needs to be addressed, as the current balance may not be suitable for all practical applications where speed is a crucial factor.
- **Scalability:** The performance of MTO-CL in high-dimensional search spaces has been effective, but its scalability to even larger or more complex problems has not been thoroughly tested. The efficiency of the algorithm in these scenarios remains uncertain and requires further investigation.
- **Generalization Across Different PV Types:** While MTO-CL has shown superior performance with specific PV modules like ST40 and SM55, its effectiveness across a broader range of solar modules and conditions has yet to be fully established. This raises questions about its generalizability and robustness in diverse settings.

Future research should prioritize optimizing the computational speed of the MTO-CL algorithm. This involves refining the algorithm’s structure and implementing more efficient computational techniques. Additionally, exploring hybrid approaches that combine MTO-CL with faster algorithms could help achieve a more balanced performance, addressing the current tradeoff between accuracy and speed.

Further investigation into algorithmic enhancements is also crucial. Potential modifications to the MTO-CL methodology could focus on reducing its computational burden while preserving or even enhancing accuracy. This could include adjusting algorithm parameters, incorporating advanced optimization strategies, or employing parallel processing to expedite computations.

Scalability testing is another key area for future research. Evaluating the performance of MTO-CL in more complex and higher-dimensional optimization problems will provide insights into its applicability for larger and more intricate systems. Understanding how the algorithm scales will be essential for determining its effectiveness in broader contexts.

Additionally, expanding the evaluation of MTO-CL to include a diverse range of PV modules and operational conditions is important. This cross-validation will help assess the algorithm’s generalizability and robustness, ensuring that it performs effectively across different types of solar panels and real-world scenarios.

Finally, exploring the integration of MTO-CL with real-time monitoring and control systems for PV installations could enhance its practical applicability. Research in this area could focus on adapting the algorithm for real-time parameter estimation and optimization, which would be particularly beneficial in dynamic environments where immediate adjustments are needed.

## 8. Conclusion

This paper has introduced the Mother Tree Optimization with Climate Change (MTO-CL) algorithm for the accurate prediction of parameters in the Three-Diode Model (TDM) of photovoltaic (PV) systems. By drawing on the foundational principles of the Mother Tree Optimization Algorithm, MTO-CL has demonstrated its capability to address complex optimization challenges within high-dimensional search spaces. The core contribution of this research lies in presenting an efficient methodology for parameter estimation in the TDM PV model, which involves nine non-linear parameters. This model is essential for capturing the intricate relationship between voltage and current in photovoltaic systems. The use of Root Mean Square Error (RMSE) minimization between simulated and experimental currents as the key cost function has proven effective for extracting these variables.

Our research has provided a thorough comparison of the MTO-CL algorithm against seven alternative optimization methods, highlighting its exceptional precision in parameter identification across various solar modules, such as the ST40 and SM55. MTO-CL consistently achieved low RMSE values ranging from 0.0025A to 0.0165A, and minimal Mean Squared Error (MSE) values from 6.2 × 10^−6 to 2.7 × 10^−4. Additionally, the algorithm effectively reduced power errors, with recorded values between 22.86 mW and 239.40 mW. These results underscore MTO-CL’s robustness and its potential as a superior choice for tackling optimization challenges in solar energy applications.

Theoretically, this study contributes to the field of photovoltaic modeling by advancing the accuracy of parameter estimation through the innovative application of MTO-CL. The algorithm’s effectiveness in navigating complex, high-dimensional optimization problems offers new insights into the potential for metaheuristic methods in enhancing PV system simulations. This research also expands the understanding of how dynamic adaptation, inspired by climate change simulations, can improve optimization outcomes in real-world scenarios.

Practically, the MTO-CL algorithm offers significant advantages in terms of precision and reliability for PV parameter estimation. Its ability to achieve globally optimal values with minimal iterations demonstrates its efficiency compared to other methods. However, it is important to note that this high level of accuracy comes at the expense of computational speed. Future work will focus on improving the algorithm’s processing time to enhance its practicality for real-time applications. Overall, the MTO-CL algorithm provides a promising tool for more accurate and effective modeling of photovoltaic systems, paving the way for better performance and optimization in solar energy applications.
